# 1318. Clinical Characteristics of Patients with Patent Foramen Ovale and Pyogenic Brain Abscess

**DOI:** 10.1093/ofid/ofad500.1157

**Published:** 2023-11-27

**Authors:** Supavit Chesdachai, Hussam Tabaja, Zachary A Yetmar, Mark Enzler, Guy S Reeder, Daniel C DeSimone, Larry M Baddour, Omar M Abu Saleh, Cristina G Corsini Campioli

**Affiliations:** Mayo Clinic, Rochester, MN; Mayo Clinic, Rochester, MN; Mayo Clinic, Rochester, MN; Mayo Clinic College of Medicine, Rochester MN, Rochester, Minnesota; Mayo Clinic, Rochester, MN; Mayo Clinic, Rochester, MN; Mayo Clinic College of Medicine, Rochester, MN; Mayo Clinic Rochester, Rochester, Minnesota; Mayo Clinic, Rochester, MN

## Abstract

**Background:**

Patent foramen ovale (PFO) can be identified in about 20-25% of the adult population and may result in variable degrees of left-to-right and right-to-left intracardiac shunting. The association between PFO and pyogenic brain abscess remains poorly understood. The current study aimed to compare the clinical characteristics and outcomes of pyogenic brain abscess in patients with and without PFO.

**Methods:**

All adults with pyogenic brain abscess seen at Mayo Clinic from 1/1/2009-12/31/2021 who underwent either transesophageal echocardiography (TEE) or transthoracic echocardiography (TTE) with agitated saline shunt (bubble) study were included.

**Results:**

A total of 222 patients developed pyogenic brain abscess during the study period; 148 (66.7%) patients were excluded due to unknown PFO status. Among 74 remaining patients, PFO was detected in 30 (40.5%) patients; 25 by TEE and 5 by TTE with bubble study (Figure 1). The prevalence of cryptogenic brain abscess (unable to identify the source of abscess) was significantly higher in patients with PFO as compared to those without PFO (53.3% versus 18.2%, p=0.002). Patients with PFO had a larger abscess size (25 mm [IQR 15.8-37.5] versus 15.5 mm [IQR 10.0-28.0], p=0.038). However, multiple abscesses were more common in the group without PFO (43.2% versus 20.0%, p=0.038). Additionally, patients with PFO had less documented bloodstream infection. There were no differences between other clinical characteristics and one-year, all-cause mortality between two groups.

**Figure:**
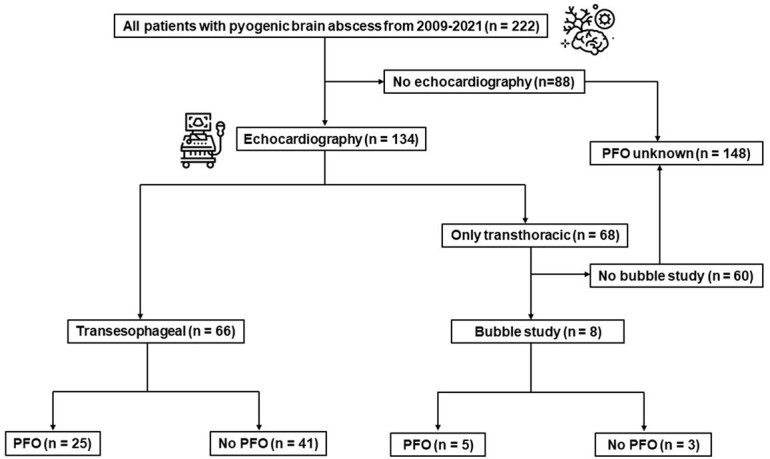

**Conclusion:**

These findings provide critical insights into the likely association between PFO and cryptogenic brain abscess; additional investigation is warranted.

**Disclosures:**

**Larry M. Baddour, MD**, Boston Scientific: Advisor/Consultant|Roivant Sciences: Advisor/Consultant

